# Secretor Genotype (*FUT2* gene) Is Strongly Associated with the Composition of *Bifidobacteria* in the Human Intestine

**DOI:** 10.1371/journal.pone.0020113

**Published:** 2011-05-19

**Authors:** Pirjo Wacklin, Harri Mäkivuokko, Noora Alakulppi, Janne Nikkilä, Heli Tenkanen, Jarkko Räbinä, Jukka Partanen, Kari Aranko, Jaana Mättö

**Affiliations:** Finnish Red Cross Blood Service, Helsinki, Finland; National Institutes of Health, United States of America

## Abstract

Intestinal microbiota plays an important role in human health, and its composition is determined by several factors, such as diet and host genotype. However, thus far it has remained unknown which host genes are determinants for the microbiota composition. We studied the diversity and abundance of dominant bacteria and bifidobacteria from the faecal samples of 71 healthy individuals. In this cohort, 14 were non-secretor individuals and the remainders were secretors. The secretor status is defined by the expression of the ABH and Lewis histo-blood group antigens in the intestinal mucus and other secretions. It is determined by fucosyltransferase 2 enzyme, encoded by the *FUT2* gene. Non-functional enzyme resulting from a nonsense mutation in the *FUT2* gene leads to the non-secretor phenotype. PCR-DGGE and qPCR methods were applied for the intestinal microbiota analysis. Principal component analysis of bifidobacterial DGGE profiles showed that the samples of non-secretor individuals formed a separate cluster within the secretor samples. Moreover, bifidobacterial diversity (p<0.0001), richness (p<0.0003), and abundance (p<0.05) were significantly reduced in the samples from the non-secretor individuals as compared with those from the secretor individuals. The non-secretor individuals lacked, or were rarely colonized by, several genotypes related to *B. bifidum*, *B. adolescentis* and *B. catenulatum/pseudocatenulatum.* In contrast to bifidobacteria, several bacterial genotypes were more common and the richness (p<0.04) of dominant bacteria as detected by PCR-DGGE was higher in the non-secretor individuals than in the secretor individuals. We showed that the diversity and composition of the human bifidobacterial population is strongly associated with the histo-blood group ABH secretor/non-secretor status, which consequently appears to be one of the host genetic determinants for the composition of the intestinal microbiota. This association can be explained by the difference between the secretor and non-secretor individuals in their expression of ABH and Lewis glycan epitopes in the mucosa.

## Introduction

Growing evidence shows that the composition and diversity of the microbiota in the human intestine can have a surprisingly strong impact on the well-being and health of the host. For example, inflammatory bowel disease (IBD) has been associated with the disturbance of the intestinal microbiota, resulting in the modulation and dysregulation of the inflammatory responses in the intestine [1]. Microbiota composition has been shown to have an effect on the energy harvest and storage of the host [2] and thus, microbiota alterations associated with obesity may have a role in weight-associated health problems.

The microbiota composition in the human intestinal tract is determined by several factors, such as host genotype, health status, age, microbial interactions, and diet [3]. Based on numerous intervention studies, there is convincing evidence for the influence of the diet on the intestinal microbiota (e.g. [4], [5]). In contrast, although growing evidence indicates that host genetic background has a significant impact on the microbiota composition in the intestine, no specific genetic factors determining the intestinal microbiota composition have been established to date. Twin studies applying plate counts, PCR-DGGE fingerprinting or DNA microarrays have shown a higher similarity in the microbiota composition between monozygotic twins than between dizygotic twins, unrelated persons, marital couples and family members [6]–[7], thus clearly pointing to a strong effect of host genetics. In the study by Turnbaugh et al. [8], the pyrosequencing analysis also showed a higher level of similarity in the microbiota composition in twin pairs than between twins and their mothers or unrelated persons, although in their study the similarity of the microbiota between monozygotic twins did not differ from that of dizygotic twins.

The human intestinal tract is colonised with highly diverse and numerous microbiota which has an established role in maintaining the intestinal homeostasis. A particularly interesting group is bifidobacteria, which comprise the predominant intestinal microbiota in infants and are abundant also in the adult population comprising up to 6% of the normal intestinal microbiota [9]. An adult intestine is typically colonised with one to four bifidobacterial species [10], *B. longum*, *B. adolescentis*, *B. bifidum* and *B. catenulatum* being the most prevalent [11], [12]. Bifidobacteria have beneficial properties, such as immunomodulatory and pathogen inhibition effects (reviewed by [13]). They also are commonly incorporated in probiotic products.

The A, B and H blood group antigens are α1,2-linked fucose containing glycans present on glycoproteins and glycolipids of erythrocytes (red blood cells) in individuals representing A, B and H blood groups, respectively. The enzyme fucosyltransferase 1 encoded by the *FUT1* gene is responsible for the synthesis of ABH antigens on erythrocytes. The ABH antigens are also expressed in mucus and other secretions, where their expression is generated by another enzyme, fucosyltransferase 2 (secretor type α1,2-fucosyltransferase) encoded by the *FUT2* gene. In ABH secretor individuals (80% of Caucasians) fucosyltransferase 2 converts type 1 N-acetyllactosamine glycan chains to H antigen, which functions as a precursor for the A, B and Lewis b antigens. Non-secretor individuals do not express active fucosyltransferase 2 enzyme due to a non-sense mutation in the *FUT2* gene and therefore they are not able to express the ABH antigens in their mucus and other secretions. The *FUT2* gene together with the *FUT3* gene encoding fucosyltransferase 3 (Lewis type α1,3/4-fucosyltransferase), is also required for the synthesis of Lewis b histo-blood group (Le a^−^b^+^) antigens in secretions. In non-secretor individuals, the *FUT3* gives rise to the Lewis a histo-blood group (Le a^+^b^−^) antigen due to non-functional fucosyltransferase 2. Lewis negative (Le a^−^b^−^) individuals have a mutation in *FUT3* gene leading to Lewis null phenotype, irrespective of the secretor status or *FUT2* gene. These mucosal ABH and Lewis histo-blood group antigens are known to serve as an energy source [14] and adhesion receptors for many microbes [15], and thus could play a role in shaping the microbiota composition of the host.

In the present study, we report that the genetic variation in the human fucosyltransferase 2 (*FUT2*) gene determining the presence of mucosal α1,2-fucosylated glycan structures, such as ABH and Lewis b histo-blood group antigens, is strongly associated with the microbiota composition, in particular that of bifidobacteria, in the human intestinal tract. We studied the association of the secretor status (determined by the *FUT2* gene) with the intestinal microbiota composition by comparing the dominant bacterial and bifidobacterial populations in faecal samples of non-secretor and secretor individuals. The denaturing gradient gel electrophoresis (PCR-DGGE) and qPCR analysis showed that the composition of the intestinal microbiota and particularly bifidobacteria was strongly associated with the host's secretor status. Secretor status determining the expression of the ABH and Lewis b glycan epitopes in the human intestine seems to be one of the host features significantly shaping the composition of bifidobacteria in the intestine.

## Results

### Blood group analysis

Fourteen of the study individuals were non-secretors and 57 secretors. Twelve of the individuals had Lewis a blood group and 48 had Lewis b blood group (Table 1). In addition, 11 individuals represented Lewis negative blood group, expressing neither Lewis a nor Lewis b antigens. For the Lewis negative samples, secretor status could not be determined by the hemagglutination assay. The secretor status determination of the Lewis negative individuals was based on the sequencing of the coding exon of the *FUT2* gene.

**Table 1:** Distribution of ABH and Lewis Blood groups in the studied individuals.

Blood group	Non-secretor (14)	Secretor(57)	All (71)
A (%)	8 (57%)	20 (34%)	28 (39%)
AB (%)	2 (14%)	9 (16%)	11 (15%)
B (%)	1 (7%)	9 (16%)	10 (14%)
O (%)	3 (21%)	19 (33%)	22 (31%)
Lewis a (Le a^+^b^−^)	12	0	12
Lewis b (Le a^−^b^+^)	0	48	48
Lewis negative^A^ (Le a^−^b^−^)	2	9	11

A Secretor status of the Lewis negative individuals was determined by sequencing the coding exon of the *FUT2* gene.

The sequencing of the *FUT2* exon showed that 9 of the Lewis negative individuals with unknown secretor phenotype turned out to be secretors and two were non-secretors, that is, they were homozygous for the 428G>A mutation leading to a *FUT2* null-allele (428G>A, se^428^, W143X, rs601338) (Table 2). All individuals with the non-secretor phenotype were homozygous for the same *FUT2* null-allele caused by the 428G>A mutation. Individuals with a secretor phenotype were either homozygous (GG) or heterozygous (GA) at the position 428 (Table 2) generating a functional *FUT2* gene. There were no discrepancies between the serological and gene level determinations of the secretor status (Table 2).

**Table 2:** Comparison of *FUT2* genotypedeterminations with secretor phenotype determinations.

FUT2 genotype based on 428G>A SNP	Phenotype			Total
	Non-secretor	Secretor	Unknown^A^	
Non-secretor (AA)	12	0	2	14
Secretor (GA)	0	27	6	33
Secretor (GG)	0	21	3	24

A Secretor phenotype could not be determined for the Lewis negative individuals by hemagglutination assay (phenotypic assay) applied here.

### PCR-DGGE gel-to-gel variation

In order to estimate the gel-to-gel variation in the PCR-DGGE analysis, we repeated 18 samples two or three times in the bifidobacterial–DGGE. The correlation of the replicate samples was 0.99 when PCR-DGGE band intensity values were used and 0.91 when the presence/absence of the bands was used, indicating that profiles of the replicate samples were highly similar and the gel-to-gel variation was low.

### PCR-DGGE of dominant intestinal bacteria

**Figure 1:**
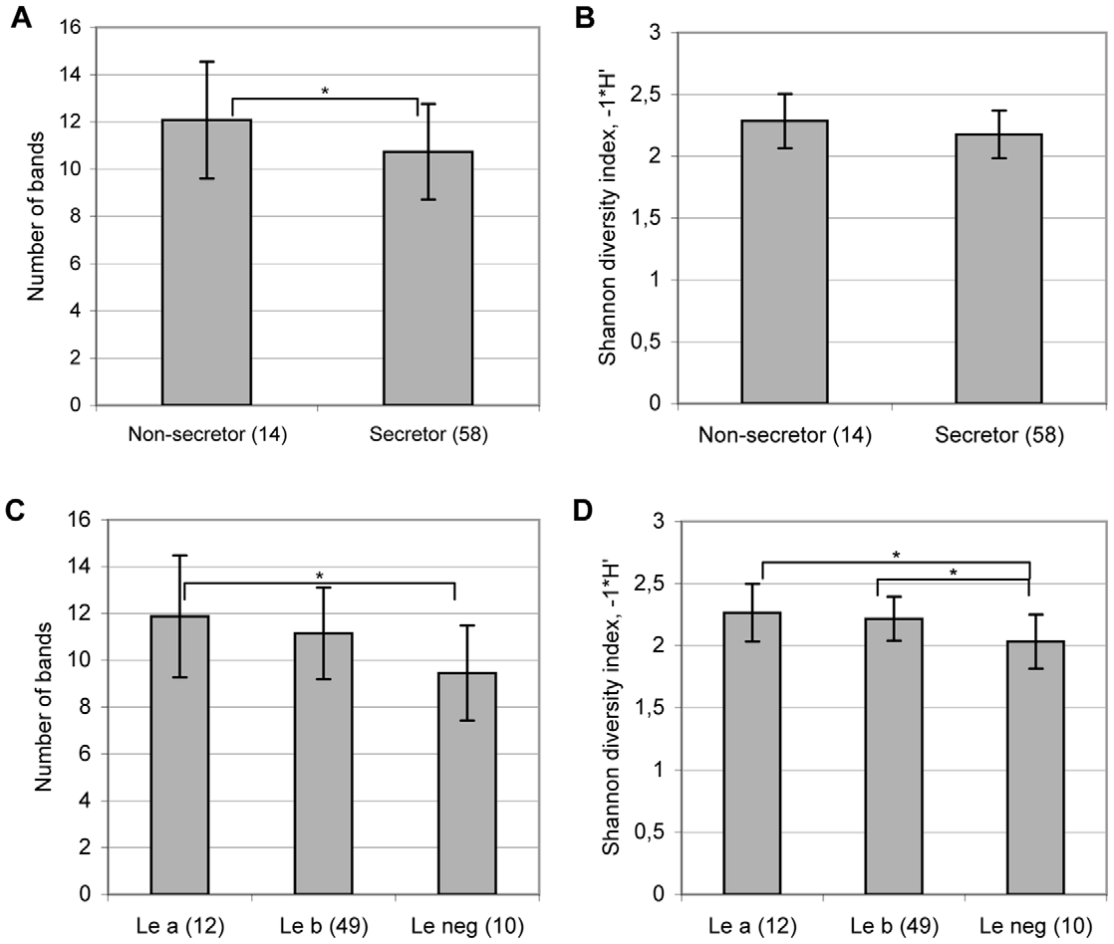
Richness and diversity of dominant bacteria in faecal samples of the non-secretor and secretor individuals (A, B) and of the Lewis a, b and negative individuals (C, D) based on the DGGE profiles. A significant difference between the groups by ANOVA: ^*^ p<0.05. In addition, a trend (p = 0.07) towards higher diversity in the non-secretor than in secretor individuals and towards higher richness in Le b individuals than in Le negative individuals was detected.

The richness (i.e. number of bands) of dominant bacteria obtained by PCR-DGGE with universal bacterial primers, was significantly higher in the non-secretor than in the secretor individuals (ANOVA; p = 0.03)(Fig. 1), showing that the non-secretor individuals had on average more bacterial genotypes over the detection limit. The bacterial diversity in the samples was measured by Shannon diversity index using the DGGE band intensity values. The Shannon diversity index showed a trend towards an increased bacterial diversity in the non-secretor individuals (p = 0.07). In addition, the individuals with the Lewis negative blood group had lower richness (ANOVA, p = 0.02) and diversity (ANOVA, p = 0.02) of dominant bacteria than the Lewis a individuals and lower diversity (ANOVA, p = 0.02) than the Lewis b individuals (Fig. 1).

Eight DGGE band positions, or “DGGE genotypes”, were statistically (Fisher's exact test, p-value between 0.0005 and 0.05) more commonly detected in the non-secretor than in the secretor individuals (Table 3). However, no clustering between the secretor and non-secretor samples was detected in the PCA of DGGE profiles of the dominant microbiota suggesting that other factors dominate the variation in microbiota.

**Table 3:** The significantly differing band positions and the incidence of bands in secretor (14) and non-secretor samples (57) by PCR-DGGE with universal bacterial primers.

Band position^A^	Detected bands	% of non-secretors	% of secretors	ANOVA	Fisher's exact test
25.20 %	22	43	28	0.02	0.05
60.20 %	19	36	25	0.01	ns^B^
56.60 %	17	64	14	ns	0.0005
39.00 %	11	36	11	0.004	0.01
42.40 %	9	29	9	0.02	0.07
47.00 %	7	21	7	0.05	ns
50.50 %	6	29	4	0.001	0.01
61.10 %	4	21	1.8	0.0002	0.03

A Only band positions, which were statistically significantly different between the groups by ANOVA or/and Fisher exact test are shown. ^B^ns = non-significant.

### PCR-DGGE analysis of intestinal bifidobacterial community

**Figure 2:**
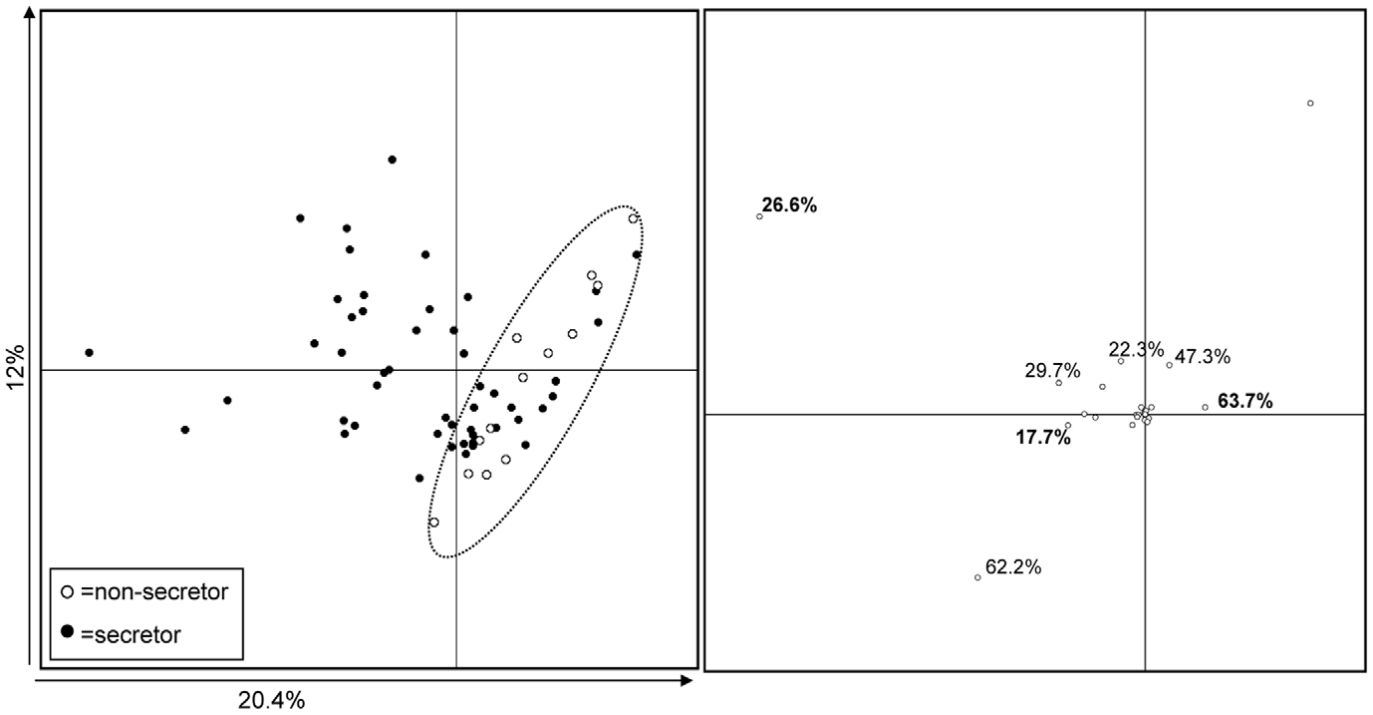
PCA plot based on the bifidobacterial DGGE profiles of faecal samples from the non-secretor (open circles) and secretor (closed circles) individuals (A) and DGGE bands contributing to the principal components 1 and 2 (B). In panel B, the numbers in bold indicate the band positions, which were significantly less commonly (Fisher's exact test, p<0.01) detected in the non-secretor individuals than in the secretor individuals (See Table 4).

The non-secretor individuals formed a separate cluster within the secretor individuals in the PCA analysis of bifidobacterial DGGE profiles (Fig. 2), indicating that the bifidobacterial population was different in the non-secretor individuals in comparison with the secretor individuals. The Lewis negative individuals did not cluster separately from the Lewis a or Lewis b individuals.

The bifidobacterial band positions that mainly contributed to the PCA clustering were 17.70%, 20.4%, 26.6%, 62.2% and 63.70% (Fig. 2). Three of these band positions (26.6%, 63.70% and 17.70%) were significantly more common in the secretor individuals than in the non-secretor individuals (Fisher's exact test, p<0.01) (Table 4). None of the 14 non-secretors had band in positions 17.70% and 63.70%. Furthermore, the band position 26.60% was clearly less frequent in the non-secretor individuals (N = 2/14; 14%) than in the secretor individuals (N = 38/57; 67%) (Table 4). These band positions were also among the most commonly detected genotypes in the secretor individuals (Table 4). Forty bands in the bifidobacterial DGGE gels, which represented 10 band positions, were excised from the DGGE gel and sequenced to identify which species/groups they represent (Table S1). With three exceptions, the band positions present in more than 10% of the samples, could be identified by sequencing (Table 4). The band position 26.60% was related to *B. adolescentis* and the position 63.70% to *B. catenulatum*/*pseudocatenulatum*, respectively (Table 4). Sequencing of the band position 17.70% was unsuccessful despite several attempts and was therefore not identified. The remaining band positions were related to *B. longum, B. bifidum,* uncultured bifidobacteria, and another *B. adolescentis* genotype (Table 4). In total, 26 band positions were detected in the Bifidobacterial-DGGE gels, 13 (50%) of these were detected at least in one non-secretor individual and all at least in one secretor individual.

**Table 4:** Identification of the bifidobacterial DGGE band positions by sequencing and the incidence of the bands in secretor (14) and non-secretor samples (57).

Best Blast hit (best cultured hit, similarity) A	Band position^B^	Detected bands	% of non-secretors	% of secretors
*B. longum*	53.5%	56	79	79
Uncultured bifidob. (*B. adolescentis*, 99%)	62.2%	41	50	60
*B. adolescentis*	26.6%	40	14	67**
not sequenced	17.7%	18	0	32**
*B. catenulatum/pseudocatenulatum*	63.7%	18	0	32**
*B. bifidum*	29.7%	17	7	28
not sequenced	20.4%	16	7	26
*B. adolescentis*	22.3%	13	14	19
Uncultured bifidob. (*B. adolescentis/ruminantium*, 98–99%)	43.8%	13	14	19
Uncultured bifidob. (*B. catenulatum*, 99%)	47.3%	9	7	14
Uncultured bifidob. (*B. adolescentis*, 99%)	55.0%	9	7	14
Uncultured bifidob. (*B. ruminantium,* 99%)	44.5%	8	7	12
Not sequenced	39.3%	7	7	11

A The similarity of the best Blast hit for a cultured strain is shown in parentheses, in cases where an uncultured bacterium was the best hit. Detailed data for the identification of each position is shown in table S1. ^B^ Only the band positions that were detected at least in 10 % of the samples are shown.** Significant differences: Band positions 26.6%, 17.70% and 63.7% were more frequently detected in secretor than non-secretor samples (Fisher's exact test, p<0.01), and in Lewis b than Lewis a samples (Fisher's exact test, p<0.01 for 17.70% and 26.60% ; p<0.02 for 63.70%).

**Figure 3:**
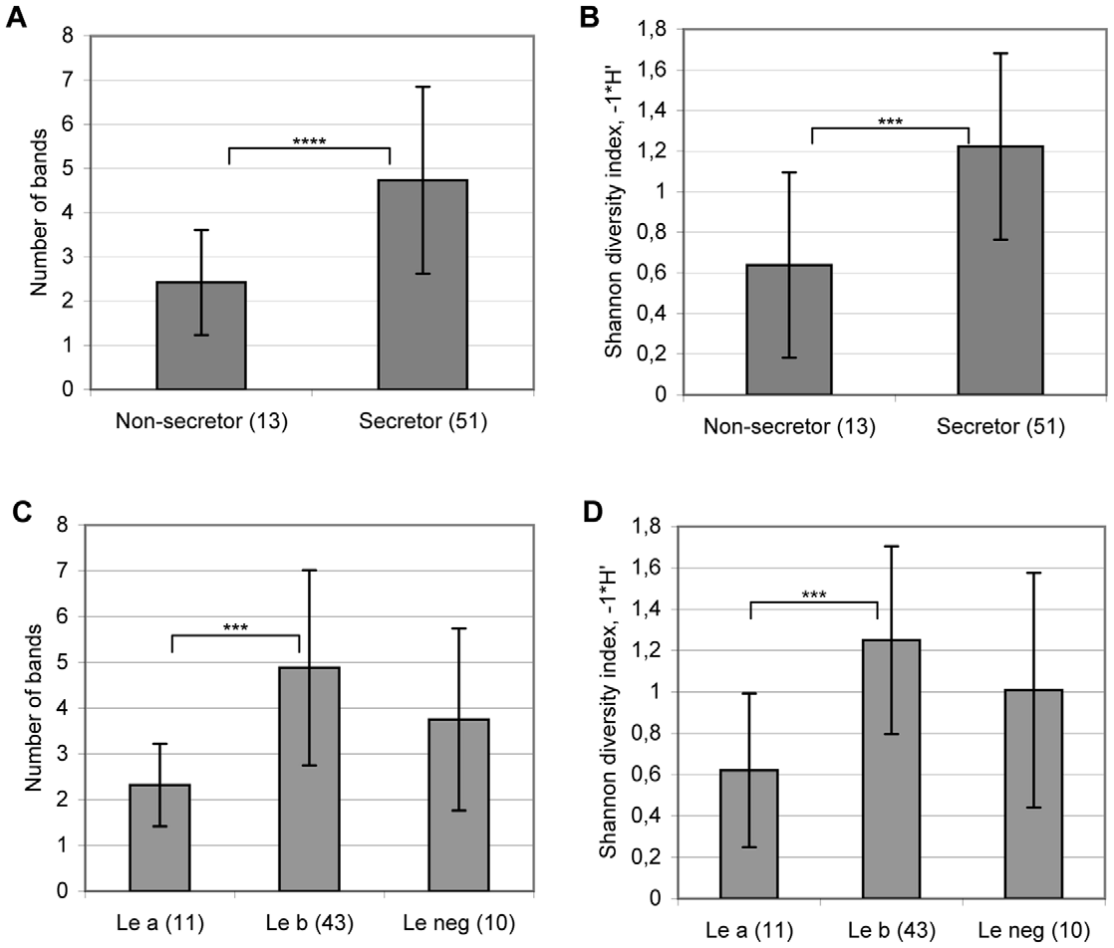
Bifidobacterial richness and diversity in faecal samples of the non-secretor and secretor individuals (A, B) and Lewis a, b and negative individuals (C, D) based on the DGGE profiles. Significant differences by ANOVA: **** p<0.0001, *** p<0.001.

The lower incidence of bifidobacterial DGGE genotypes in the non-secretor individuals was also reflected in the bifidobacterial diversity in the samples. Bifidobacterial diversity and richness in the non-secretor individuals was significantly reduced in comparison with the secretor individuals (Fig. 3). On average, the number of bands per sample in the non-secretor individuals was almost twice (1.9) as low as that in the secretor individuals. The mean number of bands was 2.5 (range from 0 to 5) in the non-secretor samples and 4.7 (range from 0 to 11) in the secretor samples. The bifidobacterial diversity and richness did not significantly differ between the Lewis-negative individuals and the Lewis a or Lewis b individuals (Fig. 3).

The inter-individual variation in bifidobacterial profiles was high in both the non-secretor and the secretor samples, as indicated by relatively low similarity values for the DGGE profiles (on average 41% between the non-secretor individuals and on average 48% between the secretor individuals).

### qPCR

**Figure 4:**
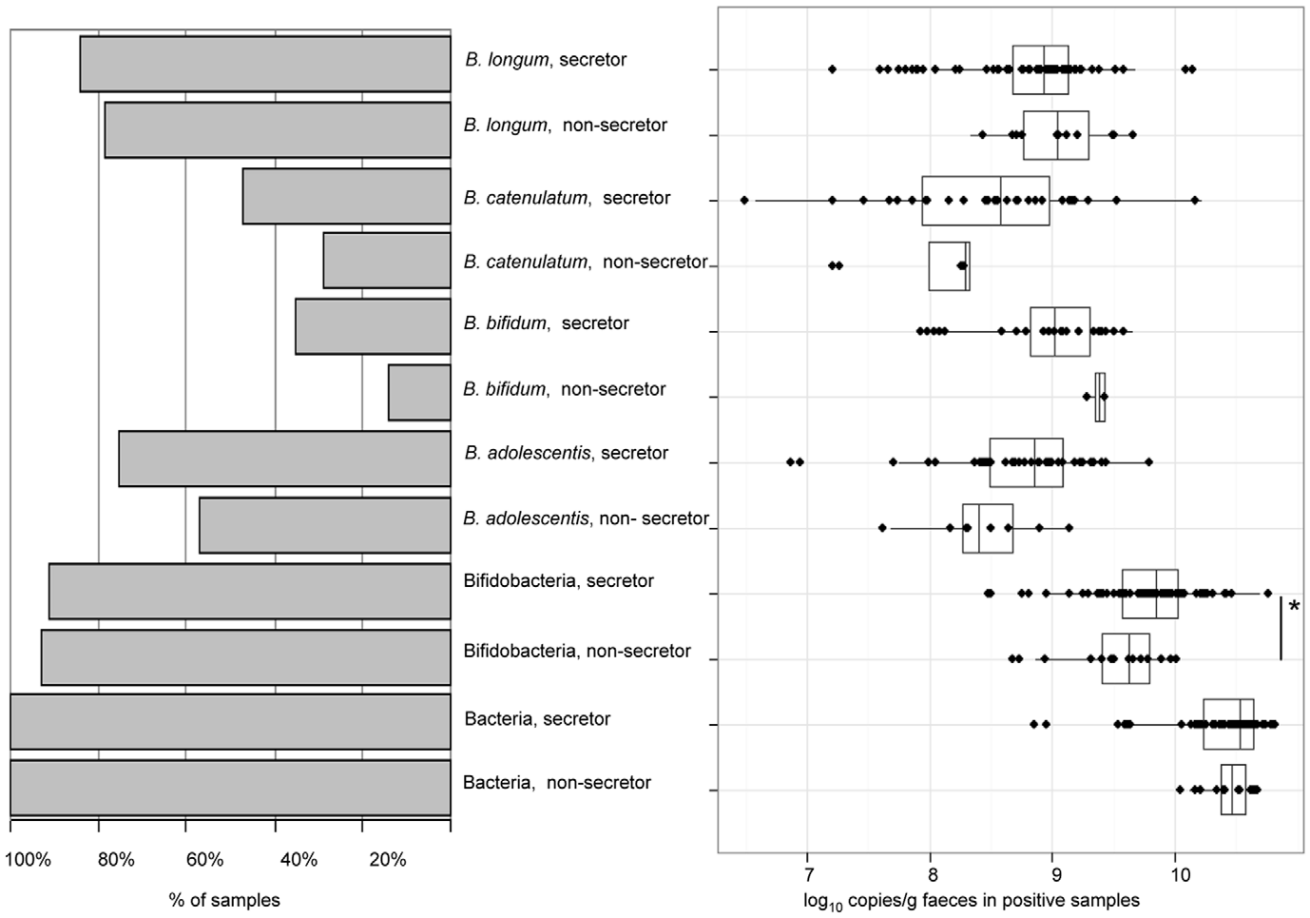
Incidence (% of samples) (left) and Box-and-Whisker plots (right) (based on log_10_ 16S rRNA gene copies per g faeces) of total bacteria, bifidobacteria and bifidobacterial groups in the faecal samples of the non-secretor and secretor individuals by qPCR. A significant difference by Wilcoxon test: ^*^ p<0.05. In addition, a trend (p = 0.06) towards higher number of the 16S rRNA gene copies of *B. adolescentis* in the secretor individuals than in the non-secretor individuals was detected.

Using a qPCR approach, bifidobacterial 16S rRNA genes were detectable in over 90% of the faecal samples. The total number of bifidobacterial 16S rRNA gene copies was lower (Wilcoxon test, p = 0.05) and fewer bifidobacterial groups were present in the non-secretor individuals in comparison with the secretor individuals (Fig. 4). All the bifidobacterial groups, *B. bifidum*, *B. longum* group, *B. catenulatum/pseudocatenulatum* and *B. adolescentis* were detected less frequently in faecal samples of the non-secretor than in samples of the secretor individuals, confirming the PCR-DGGE results. In faecal samples with detectable amounts of *B. adolescentis* group, a trend towards lower abundance of *B. adolescentis* in the non-secretor than in secretor individuals (p = 0.06) was found. The abundances of other bifidobacterial groups in samples were not significantly different. The total bacterial numbers did not differ between the secretor and non-secretor individuals (Fig. 4).

### Effect of GG homozygocity versus GA heterozygocity in *FUT2* gene 428G>A (W143X, se^428^, rs601338) SNP on the microbiota diversity

We also assessed whether homozygocity (GG) or heterozygocity (GA) of the *FUT2* gene 428G>A SNP is associated with the composition of bifidobacteria or dominant bacteria. Neither the composition nor the diversity of dominant bacteria or bifidobacteria was significantly different between the secretor individuals homozygous or heterozygous for the position 428 in the codon 143.

## Discussion

To study the association of the intestinal microbiota with the histo-blood group secretor status (defined by the *FUT2* gene), we analysed the faecal microbiota in 71 individuals, of which 14 were non-secretors. We observed that the diversity and amount of faecal bifidobacteria was considerably reduced in the non-secretor individuals. In addition to bifidobacteria, indications that the composition of dominant bacteria differed between the non-secretor and secretor individuals were discovered. Altogether these results suggest that the *FUT2* gene, which determines the presence of ABH histo-blood group glycans in mucus lining of the intestine, is a host genotypic feature significantly affecting the bacterial composition, particularly the bifidobacterial composition, in the intestine.

The secretor status determined by the *FUT2* gene was strongly associated with the bifidobacterial diversity and composition. The non-secretor individuals only had about half of the bifidobacterial diversity and richness present in the secretor individuals based on the PCR-DGGE analysis. In addition, the non-secretor individuals had significantly reduced bifidobacterial abundance in comparison with the secretor individuals as measured by qPCR. Moreover, the non-secretor individuals lacked, or were rarely colonised by, several bifidobacterial DGGE genotypes, which were related to species *B. adolescentis*, *P. catenulatum/pseudocatenulatum* and *B. bifidum,* and were common in the secretor individuals. We applied the PCR-DGGE to compare the bacterial diversity and community structure between the secretor and non-secretor individuals. The PCR-DGGE method is known to detect only the predominant part of the bacteria present in a complex sample. Bifidobacterial population is usually composed of limited number (0–4) of species [10] and thus, could be captured by the PCR-DGGE analysis with bifidobacterial specific primers. We also showed that bifidobacterial-DGGE profiles were highly reproducible. Moreover, we isolated bifidobacterial strains from the non-secretor and secretor individuals and analysed their 16S rRNA gene fragments in a DGGE gel along with faecal samples. The isolated strains corresponding to the most common (present in >10% samples) band positions in bifidobacterial DGGE gels were found (data not shown), reducing the likelihood that the detected DGGE bands originate from PCR artefacts sometimes occurring in the PCR-DGGE. In contrast to the bifidobacteria-specific DGGE, it is likely that methodological limitations hinder the interpretation of the PCR-DGGE targeted at dominant bacteria (universal PCR-DGGE). It is probable that several secretor-status associated genotypes, which are present at low levels, are missed in the PCR-DGGE using universal primers and their associations with the secretor status could, thus, not be detected. Nevertheless, our finding on the differences in the dominant microbiota between the non-secretor and secretor individuals suggests that the association between microbiota and secretor status is not limited to bifidobacteria only. It remains to be seen which other bacterial groups and species are associated with the secretor status.

The altered microbiota composition in the non-secretor individuals shown in this study may be an important factor contributing to the non-secretor disease susceptibility. Polymorphism of the *FUT2* gene, determining the secretor status, has been suggested to modulate innate immune responses and even have an evolutionary role in humans' survival during different pathogen outbreaks [16],[17]. The non-secretor phenotype has been genetically associated with increased risk for Crohn's disease [18], [19], and necrotizing enterocolitis [20]. Secretor status is also associated with the susceptibility to several infectious diseases. Non-secretors have an increased risk for urinary tract infections [21], [22] and vaginal candidiasis [23], [24], but a reduced risk for diarrhoea caused by certain genotypes of Norovirus [25]. Interestingly, many of these secretor status associated diseases, such as Crohn's disease [26], [27], urinary tract infection [28], and NEC [29] have also been connected to changes in the intestinal microbiota composition or activity. Bifidobacteria have been shown to have health promoting effects on humans [13]. Bifidobacteria or bifidobacteria-containing strain mixtures have shown promising results e.g. in the alleviation of the symptoms of irritable bowel syndrome (IBS) [30], inflammatory bowel disease [31], and diarrhoea [32], although the mechanism of action is largely unknown. Reduced bifidobacterial abundance has been connected to intestinal disorders such as irritable bowel syndrome [33] and inflammatory bowel disease [26]. Taken together, the properties of bifidobacteria and the results of this study suggest that the secretor status, by effecting bifidobacterial diversity, may also play a role in susceptibility to the diseases associated with the decreased bifidobacterial abundance in the intestine.

Metagenomics studies indicate that a considerable number of intestinal microbiota genes are involved in carbohydrate metabolism. Kurokawa et al. [34] reported that the carbohydrate metabolism genes of microbiota are enriched in the intestine (24% of genes in adults and 34% in children) in comparison with the microbiota originating from other environments, such as soil and sea. Both plant polysaccharides and host derived glycans are important energy sources for intestinal bacteria. Fucosylated histo-blood group antigens, such as the ABH and Lewis b histo-blood group antigens, are terminal epitopes of glycan chains in glycoproteins and glycolipids mediating the interaction between host and both commensal and pathogenic intestinal bacteria [35], [36]. Non-secretor individuals have null-allele of *FUT2* gene and do not express such α1,2-fucose containing glycan structures in their intestinal mucosa. Bacteria that can interact with these epitopes and compete for adhesion sites or to use them as energy sources have a better colonisation ability in secretor individuals than in non-secretor individuals (e.g. bifidobacteria in this study). Intestinal bifidobacteria, whose abundance and diversity were higher in the intestine of secretor individuals than non-secretor individuals in this study, are adapted to utilise glycans present in mucins and human milk [37]. It is known that some microorganisms secrete glycosidases capable of degrading histo-blood group antigens [14]. Comparatively small populations of human faecal bacteria produce α-glycosidases capable of degrading terminal ABH and/or Lewis groups in glycans [14]. Among them are bifidobacteria with 1,2-α-fucosidase to hydrolyse α-1,2-fucosyl linkages present in various glycans, such as the above-mentioned histo-blood group antigens [38]. Recently, Turroni et al. [39] showed, using genomic, proteomic and transcriptomic analysis of *B. bifidum* PRL2010, the existence of enzymes allowing further degradation of many core glycan chains and they concluded that the property is important for intestinal colonisation of *B. bifidum*. Such degradation of glycan cores may require the initial removal of terminal α-fucose, enabling subsequent processing of glycan chain by β-galactosidase and/or β-N-acetylhexosaminidase and endo-α-N-acetylgalactosaminidase, which catalyses the release of GalNAc from serine/threonine residues of various mucin-type glycoproteins, all of these enzymes being encoded in *B. bifidum* PRL2010 genome [39]. In addition, bifidobacteria but typically not the other common commensals, have lacto-N-biosidase degrading type 1 glycan chains, which are precursors of fucosylated histo-blood group antigens in the human intestine [40]. Therefore, it can be concluded that bifidobacteria have very specific strategies for the utilization of host glycans.

In this study we present evidence that the *FUT2* gene, which defines the secretor status and thus, the expression of the ABH and Lewis histo-blood group antigens in intestinal mucus, is one of the host genotypic features determining the composition of intestinal microbiota, particularly bifidobacterial population. We showed that bifidobacterial diversity and composition is strongly associated with the secretor status of the host. These results increase our understanding of the factors explaining inter-individual variations in intestinal microbiota composition and help us to evaluate the role of intestinal microbiota in health and disease.

## Materials and Methods

### Samples

Altogether 82 healthy adult individuals were recruited to the study from Helsinki metropolitan area, Finland. Individuals with clinically diagnosed intestinal diseases or regular intestinal disturbances were excluded from the study. The individuals had not received antibiotic therapy within two months of the faecal sampling time. Probiotic consumption was restricted one week before sampling and alcohol consumption was limited to one portion per day during three days before faecal sampling. All individuals consumed mixed diet. The study had the approval of the ethical committee of the Helsinki University Hospital and all subjects signed a written informed consent.

Both faecal and blood samples were collected from 71 subjects (7 males and 64 females). The distribution of blood groups was balanced towards the blood groups (Lewis a, Lewis negative or AB and B) rare in Finland by excluding 11 secretor individuals representing common blood groups A or O and Lewis b from faecal donation. The age of the volunteers who donated faecal samples ranged from 31 years to 61 years and was on average 44.7 years.

Faecal samples for the determination of the microbiota composition were frozen at −80°C within 5 hours of defecation. EDTA anticoagulated peripheral blood samples for blood group analysis were kept at +4°C and analysed within 24 hours. Buffy coats were extracted from citrate anticoagulated peripheral blood samples by centrifugation and stored at −80°C until DNA extraction.

### Determination of ABH and Lewis blood group and secretor status

ABO blood groups were determined by hemagglutination assay with Olympus PK 7300 according to standard blood banking practise. Lewis a and Lewis b typings were performed in tubes by monoclonal antisera (Sanquin, the Netherlands). Determination of secretor status was based on Lewis antigens. Secretor status could not be determined by phenotyping for the samples of Lewis negative individuals and their secretor status assignments were based on genotyping of the *FUT2* gene.

In addition to phenotyping, secretor status was genotyped by sequencing the coding exon of *FUT2* as described in [41] and [42]. Briefly, the *FUT2* exon was amplified with PCR and sequenced with ABI3100 in the Haartman Institute, Sequencing Core Facility (University of Helsinki, Finland) using primers described in Table 5. Individual's secretor genotype was defined as non-secretor, when the *FUT2* 428G>A SNP (se^428^, W143X, rs601338) was AA and as secretor when the *FUT2* 428G>A SNP was GA or GG. Based on the sequence analyses of the *FUT2* exon in a separate Finnish cohort consisting of 184 secretor and non-secretor individuals, no other non-secretor alleles than the *FUT2* 428G>A (se^428^, W143X, rs601338) were found in Finnish population (data not shown).

**Table 5:** Primers used in sequencing of the *FUT2* gene encoding fucosyltransferase 2.

Primer	Sequence 5′→3′	Reference
1_FUT2forward	CCATCTCCCAGCTAACGTGTCC	[41]
2_FUT2reverse	GGGAGGCAGAGAAGGAGAAAAGG	[41]
3_FUT2forward	GGGGAGTACGTCCGCTTCAC	This study
4_FUT2reverse	AGGATCTCCTGGCGGAGGTG	This study
1_FUT2PCRforward	ACACACCCACACTATGCCTGCAC	[47]
2_FUT2PCRreverse	ACTTGCAGCCCAACGCATCTT	[47]
1_FUT2SEQforward	CCAGCTAACGTGTCCCGTTTTCC	[47]
2_FUT2SEQreverse	GGCACTCATCTTGAGGGAGGCA	[47]

### DNA extraction

Total bacterial DNA was extracted from faecal samples using the FastDNA® SPIN Kit for Soil and the FastPrep® Instrument (MP Biomedicals, CA, USA) according the manufactures instruction with minor modifications. Shortly, 0.3 g faecal sample was mixed by vortexing with sodium phosphate buffer and MT buffer. Faecal slurry (1 ml) was homogenised and cells were lysed in lysing matrix E tubes with FastPrep® Instrument three times for 60 at speed setting 6.0 m/s. DNA was purified using silica based binding matrix, SPIN ™ filters, and SEWS-M wash solution. The DNA was eluted in 250 µl Dnase/pyrogen free water. Human DNA was extracted from buffy coat preparations using the QIAamp DNA Blood Mini Kit (QIAGEN Inc, CA, US). The DNA concentrations were determined with NanoDrop 1000 (Thermo Scientific, DE, USA). The extracted DNA samples were stored at − 20°C.

### PCR- DGGE

The similarity and diversity of microbiota in faecal samples of the study subjects was analysed by the PCR-DGGE. The partial 16S rRNA gene was amplified by PCR with universal bacterial primers and bifidobacterial specific primers. Amplification with universal primers U968F+GC (5′- CGCCCGGGGCGCGCCCCGGGCGGGGCGGGGGCACGGGGGGAACGCGAAGAACCTTA-3′) and U1401R (5′-CGGTGTGTACAAGACCC-3′) [43] was performed as described in Mättö et al. [44]. Bifidobacteria were amplified with primers Bif164F (5′- GGGTGGTAATGCCGGATG-3′) and Bif662R+GC (5′- CGCCCGCCGCGCGCGGCGGGCGGGGCGGGGGCACGGGGGGCCACCGTTACACCGGGAA-3′) as described in Satokari et al. [45], except elongation temperature of 72°C and Taq polymerase (Invitrogen) were used. Template DNAs were diluted to concentration 20 ng/µl for both PCRs. PCR products were obtained from all the samples with universal bacterial primers and from 64/71 samples with bifidobacterial primers. A volume of 20 µl PCR product was separated in 8% polyacrylamide gel with denaturing gradient of urea and formamide ranging from 38% to 60% (universal amplicons) or from 45% to 60% (bifidobacterial amplicons). The DGGE gels were run at 70 V for 960 mins using Dcode universal mutation detection system (Bio-Rad, CA, USA). The gels were stained with SYRB®Safe DNA gel stain (Invitrogen, Oregon, USA) for 30 mins and documented with SafeImager Bluelight table (Invitrogen) and AlphaImager HP (Alpha Innotech, South-Africa) imaging system. To estimate the gel-to-gel variation and reproducibility of the bifidobacterial PCR-DGGE method, 18 of the samples were run two or three times in different gels.

The bands were excised from the bifidobacterial DGGE gels by sterile Pasteur pipette and the DNA was eluted by incubating the gel slices in 50 µl of sterile H_2_O at +4°C overnight. The correct positions and purity of the DNA fragments eluted from the bands were checked by amplifying 1 µl of eluted DNA with primers Bif164F-Bif662R and re-running the amplified fragments along with the original samples in PCR-DGGE. Bands were sequenced in Eurofins MWG (Germany) using primers Bif164F and Bif66R without GC-rich clamp.

### qPCR

The qPCR method was applied to detect and quantify the 16S rRNA gene copies of bacteria, bifidobacteria and 4 bifidobacterial species/groups, *B. bifidum*, *B. longum* group, *B. catenulatum/pseudocatenulatum* and *B. adolescentis* in faecal samples. The primers and annealing temperature for each primer pair are shown in Table 6. Reaction mixture (25 µl) was composed of 0.3 µM of each primer (Sigma-Aldrich, UK), 1 x Power SYBR Green PCR Master Mix (Applied Biosystems, CA, USA), 4 µl faecal DNA diluted to the concentration of 1 ng/µl for bifidobacterial group/species-specific primer pair and to the concentration of 0.1 ng/µl for universal primers and bifidobacterial primers. The amplification conditions in the ABI Prism 7000 instrument (Applied Biosystems, CA, USA) were one cycle of 95 °C for 10 mins, followed by 40 cycles of 95 °C for 15 s, and appropriate annealing temperature (see Table 6) for 60 s. Melting temperature curves from 60°C to 95°C were analysed to determine the specificity of the amplification. All the samples and standards were analysed in three replicates. Standard curves from bacterial strains were constructed for each bacterial group (Table 6) by 10-fold dilutions of known concentrations of the bacterial genomic DNA (from 10 ng/µl to 0.0001 ng/µl). The Genomic DNA from the standard strains was extracted by QIAmp®DNA mini kit (Qiagen) combined with cell lysis in the FastPrep® Instrument (MP Biomedicals, CA, USA). Bacterial cells were harvested from plates, transferred to lysis tubes containing 0.1 g zirconia beads (diameter of 0.1 mm) (BioSpec products, Inc., USA) and sterile solid glass beads (diameter of 3 mm) (Sigma) in 180 µl ATL-buffer from the QIAmp®DNA mini kit. Lysis tubes were treated with FastPrep® Instrument for 60 s at speed setting 5.5 m/s. DNA concentration measurements were performed similarly to the samples (see above). GenEx Enterprise v.5.2.6.34 (MultiD Analyses AB, Sweden) was used for the analysis of standard curves and reverse quantification of the samples. The amplification efficiencies were from 93% to 98% for all the other qPCR primer pairs except for *B. bifidum* specific primers, in which amplification efficiency varied from 80% to 92% and for *B. catenulatum/pseudocatenulatum*, in which efficiency varied from 87% to 91%.

**Table 6:** Primers targeting the 16S rRNA gene, annealing temperature and strains used as standards in qPCR analysis.

Group	Primer^A^	Primer sequence 5′→3′	Anneling-T, °C	Standard strain
Bacteria	p201, p1370	GAGGAAGGNGNGGANGACGT, AGNCCCGNGAACGTATTCAC	60	*L. rhamnosus* E-96666
Bifidobacteria	qBifF, qBifR	TCGCGTCYGGTGTGAAAG, CCACATCCAGCRTCCAC	59	*B. bifidum* E-97795
*B. longum* group	BiLON-1, BiLON-2	CAGTTGATCGCATGGTCTT, TACCCGTCGAAGCCAC	62	*B. longum* E-96664
*B. bifidum*	BiBIF-1, BiBIF-2	CCACATGATCGCATGTGATTG, CCGAAGGCTTGCTCCCAAA	58	*B. bifidum* E-97795
*B. catenulatum*/*pseudocatenulatum*	BiCAT-1, BiCAT-2	CGGATGCTCCGACTCCT, CGAAGGCTTGCTCCCGAT	62	*B. catenulatum* DSM 16992
*B. adolescentis* group	BiADO-1^B^, BiADO-1b, BiADO-2	CTCCAGTTGGATGCATGTC, TCCAGTTGACCGCATGGT, CGAAGGCTTGCTCCCAGT	58	*B. adolescentis* E-981074

A References: p201, p1370 [48]; qBifF, qBIFR [49]; BiLON-1, BiLON-2, BiBIF-1, BiBIF-2, BiCAT-1, BiCAT-2, BiADO-1, BiADO-1b, BiADO-2 [50]. ^B^Two forward primers were used (BiADOg-1a and BiADOg-1b) to amplify *B. adolescentis* genotypes A and B.

### Statistical analysis

Digitalised DGGE gel images were imported to the Bionumerics program version 5.0 (Applied Maths, Belgium) for normalisation and band detection. The bands were normalised in relation to a marker sample composed of 7 common intestinal bacterial strains (for the universal PCR-DGGE) or 5 bifidobacterial strains (for the bifidobacterial PCR-DGGE). Band search and band matching using band tolerance of 1% were performed as implemented in the Bionumerics. The bands and band matching were manually checked and corrected. Samples with no amplification in the bifidobacterial PCR-DGGE were excluded from the analysis. Similarity of the bifidobacterial profiles was calculated as implemented in Bionumerics, version 5. Matrices based on band intensities and presence /absence of bands were exported from Bionumerics and used for calculation of Shannon diversity indexes in Microsoft Excel. Shannon diversity index, H', was calculated using equation H' = Σpiln(pi), where pi was proportion of each species (i.e. DGGE band intensity) in the sample. The richness was calculated as a number of detected bands in the DGGE profile of the sample. Principal component analysis (PCA) based on the band intensities was calculated as implemented in Bionumerics, version 5.0. Other statistical analyses (ANOVA, Fisher exact test and two-sample Wilcoxon test) were computed with statistical programming language R, version 2.10.1. (http://www.r-project.org/). Gel-to-gel variation was measured by comparing the DGGE profiles of the samples run in different gels. The DGGE profiles based on either band intensities or presence/absence of bands were used for calculation of the Pearson correlation between replicate samples using statistical programming language R, version 2.12.0.

The sequences retrieved from the DGGE bands were trimmed and aligned by ClustalW [46]. The closest relatives of the sequences were searched using Blast (http://blast.ncbi.nlm.nih.gov/Blast.cgi) and NCBI nr database. Distance matrix of the aligned sequences was used to compare the similarity of the sequences. The accession numbers of the sequences are FR775384-FR775395.

## Supporting Information

Table S1 The best Blast hits of the sequences derived from bifidobacterial DGGE bands
(DOC)
