# Supplementary material for: Secretor Genotype (FUT2 gene) Is Strongly Associated with the Composition of Bifidobacteria in the Human Intestine
Source: PLoS One. 2011 May 19;6(5):e20113. doi: 10.1371/journal.pone.0020113 (PMC3098274; doi:10.1371/journal.pone.0020113)
Supplement: Table S1 — The best Blast hits of the sequences derived from bifidobacterial DGGE bands (DOC) [file pone.0020113.s001.doc]

**Table S1.** The best Blast hits of the sequences derived from bifidobacterial DGGE bands.

| Band position | Representative sequence (# of sequences)^A^ | Closest relative (closest cultured relative) ^B^ | Similarity, gaps, coverage for closest relative [closest cultured relative] | # of bands |
| --- | --- | --- | --- | --- |
| 53.5% | 090626B12 (8) | *B. longum* JCM 11660^D^ | 475/475 (100%), 0/475, 100% | 63 |
| 62.2% | 090626B31 (7) | Uncultured bacterium clone L039 (*B. adolescentis* BBMN23) | 479/479 (100%), 0/479, 100% [478/479 (99%), 0/479, 100%] | 45 |
| 26.6% | 090626B15 (5) | *B. adolescentis* ATCC 15703^D^ | 479/479 (100%), 0/479, 100% | 43 |
| 63.7% | 090626B22 (2) | *B. pseudocatenulatum* JCM 7041^E^ | 478/478 (100%), 0/478, 100% | 20 |
| 22.3% | 090626B11 (1) | *B. adolescentis* ATCC 15703^D^ | 480/480 (100%), 0/480, 100% | 16 |
| 29.7% | 0090626B6 (4) | *B. bifidum* JCM 7004^D^ | 478/478 (100%), 0/478, 100% | 16 |
| 43.8% ^B^ | 090626B17 (4) | Uncultured bacterium clone B643( *B. ruminantium* JCM 8222) | 460/460 (100%), 0/460, 100% [455/457 (99%), 1/457, 99%/] | 15 |
|  | 090626B30 (1) | Uncultured *Bifidobacterium* sp. clone HM28 (*B. adolescentis* strain BBMN23) | 479/479 (100%), 0/479, 99.8% [475/480 (98%), 2/480, 100%] |  |
| 55.0% | 090626B18 (2) | *Bifidobacterium* sp. Eg1 (*B. adolescentis* ATCC 15703^C^) | 479/479 (100%), 0/479, 100% [465/468 (99%), 2/468, 97%] | 11 |
| 44.5% | 090626B36 (1) | Uncultured *Bifidobacterium* sp. clone WWTP_SFA25 (*B. ruminantium* JMC8222) | 477/479 (99%), 0/479, 100% [454/456 (99%), 1/456, 95%] | 9 |
| 47.3% | 090626B8 (1) | *Bifidobacterium* sp. h12 (*B. pseudocatenulatum* JCM 7041) | 478/478 (100%), 0/478, 99.8% [477/479 (99%), 2/479, 100%] | 9 |
| 45.7% | 091209B19 (4) | Uncultured *Bifidobacterium* sp. clone HM28 (*B. adolescentis* strain BBMN23) | 479/479 (100%), 0/479, 99.8% [475/480 (98%), 2/480, 100%] | 4 |

^A^ For each cluster of identical sequences, a representative sequence was searched in blast and submitted to the EMBL database. ^B^ When best hit was uncultured or classified only at species level, best hit to cultured strain or stain identified at genus level is shown in parentheses. ^C^ The sequence similarity of the bands in position 43.8% was 98%. ^D^ Equally good hit for several strains of the same species were. ^E^ Equally good hit for several strains of *B*. *pseudocatenulatum* and *B*. *catenulatum*.
